# Endoscopic Sleeve Gastroplasty (ESG) Versus Laparoscopic Sleeve Gastroplasty (LSG): A Comparative Review

**DOI:** 10.7759/cureus.41466

**Published:** 2023-07-06

**Authors:** Basil N Nduma, Kelly A Mofor, Jason Tatang, Loica Amougou, Stephen Nkeonye, Princess Chineme, Chukwuyem Ekhator, Solomon Ambe

**Affiliations:** 1 Internal Medicine, Merit Health Wesley, Hattiesburg, USA; 2 Gastroenterology, Texas Tech Paul L Foster School of Medicine, El Paso, USA; 3 Gastroenterology, Sam Houston State University, Huntsville, USA; 4 Gastroenterology, School of Natural Sciences and Mathematics, University of Texas at Dallas, Richardson, USA; 5 Health & Biomedical Sciences, University of Texas Rio Grande Valley, Edinburg, USA; 6 Gastroenterology, University of Texas at San Antonio, San Antonio, USA; 7 Neuro-Oncology, New York Institute of Technology, College of Osteopathic Medicine, New York, USA; 8 Neurology, Baylor Scott & White Health, Mckinney, USA

**Keywords:** body mass index, comorbidity, mortality rate, quality of life, bariatric surgery, adverse events, efficacy, safety, laparoscopic sleeve gastroplasty, endoscopic sleeve gastroplasty

## Abstract

Obesity is one of the most debilitating conditions. In a quest to mitigate disease severity, various interventions have been proposed, with endoscopic sleeve gastroplasty (ESG) and laparoscopic sleeve gastroplasty (LSG) being among the recent interventions that have received growing attention. This systematic review sought to conduct a comparative analysis regarding the efficacy, effectiveness, and safety of both interventions.

The study involved a systematic review in which key search engines were used to select articles documented and published in the last decade. The articles for inclusion were those existing as peer-reviewed studies touching upon the aforementioned subject, with both controlled and uncontrolled trials included. Furthermore, there was the implementation of the Preferred Reporting Items for Systematic Reviews and Meta-Analyses (PRISMA) protocol that governs systematic reviews, in which the article selection process entailed four key procedures in the form of identification, screening, determining eligibility, and the inclusion process.

In the findings, the selected articles documented mixed outcomes, but a common denominator was that the safety profile of ESG tends to be superior to that of LSG due to the observations that ESG comes with fewer adverse events such as gastroesophageal reflux disease (GERD) and severe nausea and vomiting. However, the majority of the studies contended that LSG proved superior to ESG in terms of effectiveness and efficacy. Hence, individuals with mild-to-moderate obesity are more likely to benefit from ESG, but those with severe obesity whose goal is to achieve long-term weight management might benefit more from LSG. In conclusion, the management of obesity and the decision to employ ESG or LSG ought to be patient-centered and dictated by factors such as patient preferences, safety, and the sustainability of the devised plan of care.

## Introduction and background

Obesity occurs in individuals when the body mass index (BMI) equals or exceeds 30  kg/m2. The multifactorial and chronic condition is marked by abnormal weight gain arising from an excessive accumulation of adipose tissue [1]. The implication is that obesity is one of the fastest-growing global health burdens, with billions of dollars spent on the management of bariatric patients [2]. What is worth remembering is that the effective management of obesity has been avowed to be achieved mostly through a multidisciplinary approach, such as the treatment and management of obesity. Particularly, one of the techniques that has been avowed to yield promising outcomes is the surgical intervention option, with a particular emphasis on severe obesity, because the approach comes with long-term improvements in the quality of life, comorbidities, and weight loss, yielding an overall decrease in patient mortality [3]. In the United States, when annual bariatric surgical procedures are considered, laparoscopic sleeve gastroplasty (LSG) accounts for as many as 59.4% of the procedures, proving the most dominant bariatric intervention [1]. Indeed, LSG entails resecting the stomach’s fundus and also the gastric greater curvature via the implementation of a partial vertical gastrectomy aimed at achieving gastric tubulation. Some of the outcomes associated with this procedure include improved comorbidities and quality of life, as well as effective loss of body weight [4]. However, it is imperative to note that despite these beneficial effects linked to LSG implementation, the procedure has been documented to come with chronic and acute complications post-operation, including gastric fistulae, leakage, and bleeding, eventually discouraging patients due to the perception that the approach is less desirable [5].

Recently, bariatric endoscopic approaches have emerged, and they have been observed to promise otherwise repeatable, more cost-effective, and less invasive approaches concerning obesity treatment [1]. Endoscopic sleeve gastroplasty (ESG) is one specific example of bariatric endoscopic techniques that have evolved. Through ESG, there is gastric tubularization, in which full-thickness sutures are placed in top-to-bottom directions in triangles, stretching to the gastric fundus from the gastric angulus. Notably, a part of the fundus itself and also the pyloric antrum area are preserved [6]. Important to remember is that the ESG method limits the amount of food introduced into an individual’s stomach as well as the number of calories that the individual could consume. In the literature, therefore, ESG has been observed to be more likely to achieve outcomes similar to those that would be realized if surgery were embraced [7]. However, it is important to remember that scientific evidence documenting ESG-related outcomes remains dire even at a time when the procedure has been perceived to come with fewer adverse events.

Indeed, ESG’s creation saw the procedure evolve as an otherwise more cost-effective and less invasive endoscopic alternative that could be used in place of LSG, yet extensive comparative studies are yet to be established. The implication is that comprehensive systematic reviews and meta-analyses combining data touching on both procedures have yet to achieve adequacy, emphasizing the unmet need. The main aim of the current study lies in the comparison of the safety and efficacy of ESG and LSG, conducted from a systematic review perspective. The motivation of the investigation is to seek to give insight into some of the parallels that could be drawn between the two procedures in terms of their beneficial effects and any associated adverse events, thereby eventually increasing the understanding of their implications for future healthcare services extended to patients presenting with obesity in clinical environments. For the same population that has obesity, whether the two approaches could be employed interchangeably is an interesting phenomenon worth clarifying via the current investigation.

## Review

Materials and methods

In this study, the Preferred Reporting Items for Systematic Reviews and Meta-Analyses (PRISMA) protocol, which governs systematic reviews, was implemented. From a systematic review approach, the study considered search strategy as the initial parameter on which to focus. Some major databases or search engines were relied upon in a quest to access and use the respective scholarly articles or studies deemed appropriate and relevant to the subject being investigated. Some of the specific databases from which data were established were Excerpta Medica Database (EMBASE), Medical Literature Analysis and Retrieval System Online (MEDLINE), PubMed, and Clinicaltrials.gov. To arrive at relevant articles, there was also the incorporation of keywords, which included "ESG", "LSG", "safety", "efficacy", "adverse events", "bariatric surgery", "quality of life", "mortality rate", and "comorbidity". Abstracts that were accessed for different articles were also used to inform the decision to include or exclude them. Prior to this decision, there was also a screening of such abstracts to determine their relevance to the given topic.

With the search criteria in place, there was the aspect of the inclusion criteria. Here, the articles to be included in the systematic review needed to be those that had been developed in the past decade. The intention was to achieve a state of recency, hence relevance to the current state-of-the-art in obesity treatment and management. With different databases relied upon, the inclusion criteria entailed further de-duplication practices to mitigate potential redundancy, touching on articles authored by the same researchers but appearing in various search engines. For potentially redundant articles and redundant information, the authors would arrive at a consensus to exclude such articles.

The inclusion criteria further held that the articles to be used in this review needed to be those that had been conducted in the form of a comparative analysis pitting ESG versus LSG’s efficacy, effectiveness, and safety, having also centered on individuals diagnosed with obesity. Hence, the intervention or experimental groups that the selected studies needed to have focused on were patients diagnosed with obesity, with specific interventions entailing ESG and LSG and the outcome variables being comorbidities, quality of life, mortality rates, and adverse events. In situations where the selected studies might have had both experimental and control groups, those that were included in the review needed to have been individuals on a placebo with no active therapy. In cases where baseline comparisons were available or used, such studies would also be included in the review, although they would not necessarily have had control groups. With outcome variables focused upon further, it can be noted that the studies that were included were those reporting evaluable data concerning ESG and/or LSG implementation, as well as the implications for obesity severity. To achieve data saturation, both uncontrolled and controlled trials were included.

The methodological consideration focused further on the process of extracting data. Here, reviewers engaged in the independent extraction of data from the literature. In the case of disagreements in the eventual themes and inferences that the respective reviewers established, a consensus was used to address them. Still, considering the process of extracting data, some of the factors or variables that each reviewer was prompted to report included the gender of the participants in the selected studies, any blinding factors, the durations of the investigation, the age of the participants, and whether both experimental and control groups were present. Similarly, the reviewers were prompted to report the articles’ authors, years of research and article publication, the intervention type (whether ESG, LSG, or both), the research context, and the comparator used. The motivation for allowing the reviewers to report these various attributes was to provide room for drawing themes and inferences as needed, as well as pave the way for more comprehensive comparative analyses within and between articles, hence making informed conclusions concerning the efficacy and safety of ESG versus LSG.

**Figure 1:**
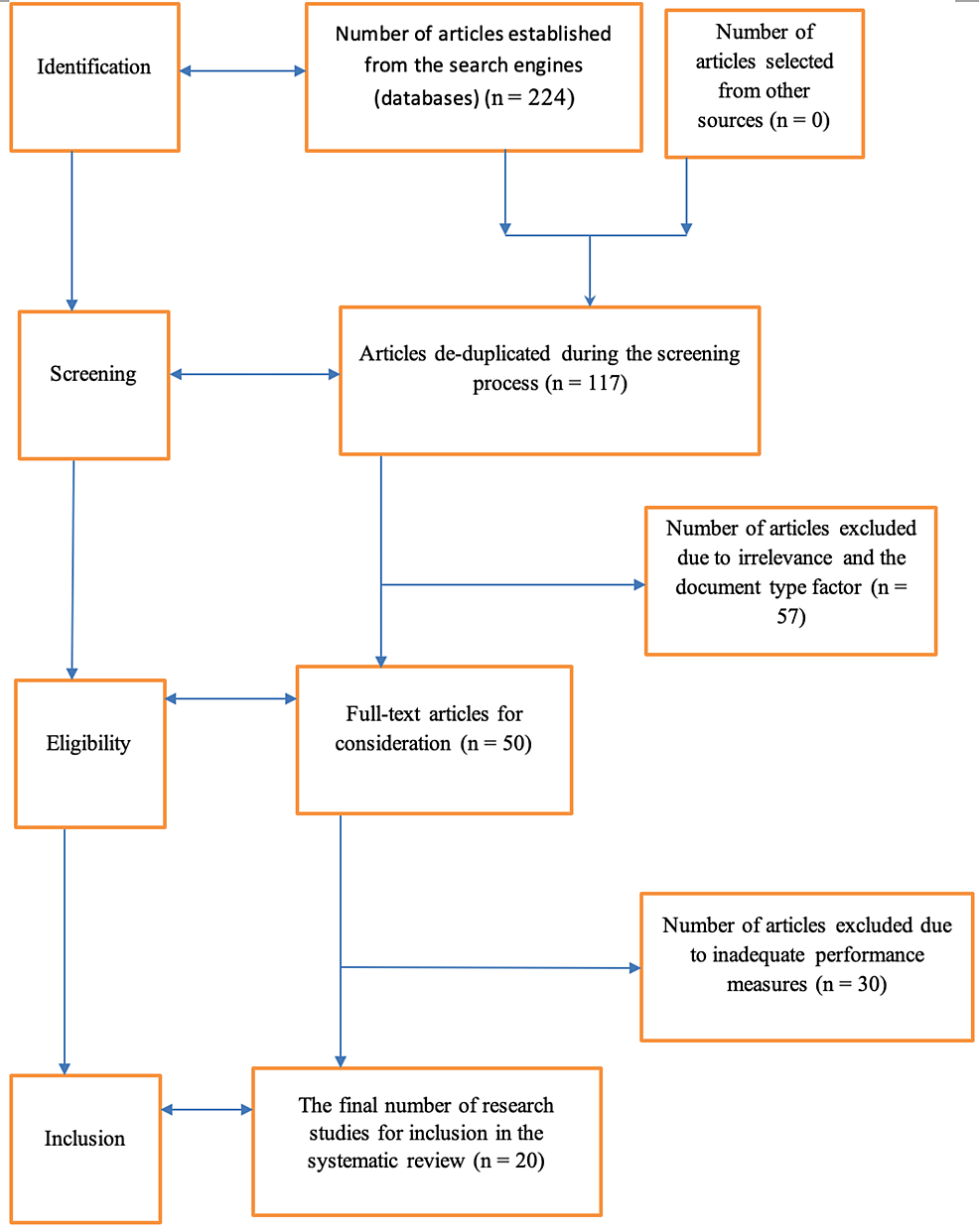
A flow diagram of the PRISMA protocol illustrating the article inclusion and exclusion procedures for the study PRISMA: Preferred Reporting Items for Systematic Reviews and Meta-Analyses

Important to remember is that further emphasis was put on the quality of the results being reported about ESG and LSG in different studies. At this point, the emphasis was on comprehensive evaluations of the investigations to confirm outcome quality, a procedure that was realized through the utilization of external reviewers who sought to determine the presence of biased reporting in the selected studies. Some of the specific forms of bias that the independent reviewers sought to unearth and determine the eligibility of the given articles for or against inclusion included attrition bias, allocation concealment, performance bias, detection bias, reporting bias, and sequence generation. Hence, any risk associated with any given article would be deemed high, low, or unclear. In the case of dissimilarities between reviewers, a corresponding author would be used to mitigate the same. The figure below indicates a summary of the PRISMA protocol that was used to guide the way in which the articles were identified and screened to discern any eligibility, upon which the final number of articles was arrived at, as informed by the inclusion and exclusion criteria described in this methodological section (Figure 1).

Results

Table 1 below outlines all of the 20 studies included in the literature review.

**Table 1:** An outline of the included studies and their findings LSG: laparoscopic sleeve gastroplasty; ESG: endoscopic sleeve gastroplasty; GERD: gastroesophageal reflux disease; UTI: urinary tract infection

Studies	Results	Clinical implications
Marincola et al. (2021) [1]	This investigation revealed a moderate superiority of LSG compared to ESG. However, there was no vivid difference in safety between the two procedures, with ESG observed to be an otherwise acceptable, repeatable, reversible, and less-invasive intervention when implemented in obese patients diagnosed with a condition of mild severity.	The implication for future clinical practices is that reaping the beneficial effects with which ESG tends to be associated might be more vivid when the procedure is applied to people with obesity of moderate severity.
Kheirvari et al. (2020) [2]	Aimed at discerning the safety and efficacy of ESG, this study established that some of the associated complications include leakage, nutrient deficiencies, and bleeding, with pre-operative complications affirmed to be dependent on ethnic and gender disparities within populations to which the population could be applied.	The study sensitized audiences to the need to consider gender and ethnic factors when making decisions for or against ESG implementation, having established that the safety of the procedure rests with the aforementioned factors.
Boškoski et al. (2020) [3]	Concerning the efficacy of ESG, this study revealed the mean percentages of total body weight loss and excess weight loss as 20.4% and 44.2%, respectively, leading to the conclusion that compared to LSG, ESG comes with satisfactory short-term benefits for patients diagnosed with obesity, hence efficacy and safety.	The study increased understanding of the importance of considering redoing ESG as a safe and effective intervention. However, an area requiring further investigation is the disease severity level to which the procedure could be applied.
Storm et al. (2019) [4]	On the one hand, LSG was affirmed to be effective, safe, and technically simple relative to weight loss. On the other hand, the procedure was documented to come with increased costs of healthcare utilization due to several associated serious adverse events as well as an increase in de novo or worsened GERD prevalence. Furthermore, the study, focusing on LSG’s safety, suggested that the procedure comes with long-term risks of esophageal adenocarcinoma and Barrett’s esophagus.	With LSG’s associated limitations, especially on the part of patients presenting with lower BMI, the study led to the inference that compared to LSG, ESG has evolved as an otherwise attractive endoscopic alternative to surgery because it is minimally invasive, comes with superior therapeutic benefits, and seeks to reflect effective obesity management procedures that target the gastrointestinal tract of persons not wishing or who may not qualify for bariatric surgery.
Sartoretto et al. (2018) [5]	At six months, the efficacy of ESG was ascertained, and between three centers, multivariable analytical outcomes suggested that the key predictors of changes in weight among patients included a lack of previous endoscopic bariatric surgery, a greater baseline body weight, and male sex.	The study increased the understanding that ESG is a safe, reproducible, and effective approach to safe weight loss, hence the need for its widespread adoption in clinical environments.
Fayad et al. (2019) [6]	With 83 LSG and 54 ESG patients selected based on the variables of BMI, sex, and age, at six months of follow-up, it was noted that the percentage of total body weight loss compared to the baseline values was much lower within the ESG group than in the LSG group, but the beneficial aspect linked to ESG was found to be profound in terms of relatively fewer adverse events. In the ESG group, too, the likelihood of GERD onset was noted to be significantly lower than in the case of patients exposed to the ESG procedure.	At this point, the authors inferred that ESG exists as a same-day and minimally invasive intervention, and, at six months of follow-up, although it achieves less weight loss compared to LSG, the intervention is less likely to cause adverse events and new-onset GERD. Hence, although the effectiveness of ESG was observed to be relatively inferior to LSG, its safety proved superior to LSG, having been linked to fewer adverse events.
James & McGowan (2019) [7]	Motivated by the need to uncover some of the safety concerns likely to be associated with the ESG procedure, this study inferred that transfundic sutures are the main risk factor likely to yield adverse procedural events during the implementation of the intervention, with a particular emphasis on the formation of perigastric abscesses.	The study increased the understanding that in the fundus, suture placement is worth avoiding, thereby mitigating the risks of possible serious adverse events following ESG implementation.
Mohan et al. (2020) [8]	ESG was found to appear to be a key and effective alternative option to LSG regarding obesity treatment, evolving as a reversible procedure whose hospital length of stay was observed to be shorter, procedure time faster, and safety profile better. However, superior loss of total weight was associated with LSG at one year compared to the case of ESG.	The study pointed to the long-term efficacy of LSG but also revealed the safety aspect of ESG, pointing to the need to consider additional factors such as cost-effectiveness and long-term health outcomes when making decisions for ESG or LSG implementation.
Yoon & Arau (2021) [9]	In this study, the safety profile linked to ESG was found to be superior compared to LSG’s associated safety profile. Therefore, ESG was observed to yield reductions in obesity-related comorbidity risk, having reduced the HbA1c level significantly. Furthermore, ESG yielded notable reductions in the risk of fibrosis and hepatic steatosis, triglyceride level, and systolic blood pressure, with improved quality of life also noted.	The implication for future clinical environments is that, compared to LSG, ESG could serve as a safe intervention, qualifying as an alternative treatment to the former (LSG).
Carr et al. (2022) [10]	Applied as weight loss treatment modalities, both LSG and ESG procedures were documented to be effective and safe on the part of obese adults, with the effectiveness and safety found to be moderated by the variable of multidisciplinary support provision. However, fat-free mass was found to be maintained in the ESG group at six months, but at 12 months post-procedure, both the LSG and the ESG groups lost fat-free mass. Also, patients linked to the LSG procedure were observed to experience large and significant improvements concerning the quality of life related to weight.	To ascertain these findings, the study pointed to the need for future investigations to focus on alternative populations with varying demographic features such as age, gender, and ethnicity. Also, despite the safety and effectiveness of both procedures noted, LSG came with mild-severe adverse events at 27%, while ESG came with mild-moderate adverse events at 25%. Thus, ESG proved safer than LSG.
Polese et al. (2022) [11]	In high-risk surgical patients with an initial BMI of less than 40, the study established ESG as a safe and effective approach. However, compared to LSG, ESG was avowed to be less effective, despite being significantly safer. The rate of adverse events in LSG was documented to be as high as 16.9%, while that of ESG’s associated adverse events was observed to stand at 5.2%.	The authors concluded that ESG is superior to LSG in terms of safety but less effective compared to the latter, pointing to the need for longer follow-up studies with larger sample sizes to ascertain such outcomes.
Marshall et al. (2022) [12]	In this study, findings suggested very little evidence suggesting differences in predictors of ESG and LSG’s safety and effectiveness or efficacy, including outcome variables of weight-related quality of life, body fat percentage, and rates of comorbidities.	The results point to the need for further clinical investigations to determine ways in which patient-centeredness could be achieved and also ways through which more informed decisions could be made when seeking to implement ESG or LSG.
Ibrahim Mohamed et al. (2022) [13]	ESG was documented to come with a lower complication rate, and the length of the hospital stay was found to be shorter post-procedure, but LSG was associated with greater BMI reduction as well as the mean percentage of the total body weight loss at one year compared to ESG.	Similar to the majority of other studies, this study confirmed ESG’s superiority in terms of safety and LSG’s superiority in terms of effectiveness or efficacy.
Wang & Chen (2020) [14]	Whereas ESG was observed to yield satisfactory efficacy concerning the factor of weight loss, it was still found to be inferior to the LSG approach. However, it was in the ESG group that the risks of adverse events were documented to be lower within 12 months of follow-up. For patients associated with poor adherence to behavioral interventions, ESG was associated with better weight control outcomes than LSG.	Some of the factors explaining the effect of decreased weight loss would be worth investigating further, as would determining some of the factors that could cause weight regain in the ESG group.
Fayad et al. (2022) [15]	The authors indicated that when ESG is implemented, the procedure comes with a significantly shorter length of stay and a lower morbidity rate than LSG, as well as a lower rate of adverse events. In the LSG group, it was noted that de-novo GERD is likely to arise at higher rates than in the ESG group.	The study suggested that ESG comes with better short-term safety outcomes or a superior safety profile than LSG, hence the need to consider this factor when embracing patient-centeredness and making decisions for or against ESG or LSG implementation.
Lavín-Alconero et al. (2021) [16]	When it comes to the remission of hepatic alterations and weight reduction, LSG was observed to be more effective than ESG, but the procedure would come with chronic and acute complications post-operatively, coming at a time when the safety profile of ESG was avowed to be far superior, evolving as a more cost-effective and less invasive approach.	In terms of patient safety, the study demonstrated that ESG is a promising novel endoscopic technique whose effectiveness mostly applies to obese patients with mild-to-moderate severity.
Qureshi et al. (2023) [17]	Whereas the total body weight loss in the ESG group was documented to be lower than that in the LSG group, the rate of adverse events in the ESG group was established to be more promising.	With LSG associated with complications such as significant nausea and vomiting, UTI, wound infections, prolonged postoperative ileus, pulmonary embolism, visceral herniation, and peri-gastric leak, the implication is that the quest to benefit from the procedure’s associated efficacy needs to be accompanied by the consideration of such safety concerns.
Singh et al. (2020) [18]	ESG was documented to be minimally invasive and a reproducible procedure across the world, coming with a favorable safety profile and effective weight loss outcomes because the procedure is reversible and does not require abdominal incisions, but durable and substantial weight loss was associated with LSG.	The implication is that the severity of obesity and the intervention goals ought to inform whether to use ESG or LSG therapy.
Jalal et al. (2020) [19]	Compared to LSG, ESG was found to yield short-term weight loss, but it was associated with fewer complications. After 12 months, weight loss outcomes in the ESG group were avowed to plateau.	Whereas ESG was established to be a minimally invasive approach with fewer complications, its future uptake will need to be informed by the intervention goal concerning the sustainability of weight loss in obese patients.
Beran et al. (2022) [20]	Whereas there was the achievement of clinically adequate mid-term and short-term weight loss in the ESG group, it was lower when compared to the case of the LSG group. However, the ESG group experienced fewer adverse events such as GERD.	The authors came to the understanding that ESG has a stomach-sparing nature, hence the associated acceptable safety profile. In patients diagnosed with mild-to-moderate obesity, therefore, ESG is a therapy, intervention, or procedure worth comparing ahead of LSG. If the goal is to achieve higher weight loss, however, the study pointed to the need to consider LSG despite its associated number or incidence of adverse events that exceed those experienced in the ESG group.

In the literature on the comparative analysis between ESG and LSG, with a particular emphasis on the safety and efficacy of the two procedures, one article with participants involving patients diagnosed with obesity whose BMI was in the range of 30 kg/m2 to 40 kg/m2 and the minimum follow-up duration was 12 months reviewed 16 studies, constituting 2,188 patients in total, with those exposed to LSG being 1,429 and those undergoing ESG standing at 759. In the results, the mean excess weight loss percentage stood at 80.32% for the LSG group and 62.20% for the ESG group; hence, a value of 18.12% was obtained as the absolute difference, with LSG exhibiting a moderate superiority compared to ESG, which was less invasive and preferred for patients with mild to moderate obesity [1].

In another study, a comparative analysis of ESG versus LSG was conducted from a case-matched perspective at a single academic institution. The follow-up duration was between one and six months post-procedure, with the main dependent variable being the percentage of total body weight loss as a predictor of the efficacy of the procedures. Other variables that were investigated include new-onset gastroesophageal reflux disease (GERD) and adverse events, thereby informing the safety [6]. With 83 LSG patients and 54 ESG patients on the focus list at baseline, the proportion of those with GERD was 25.3% in the LSG group and 16.7% in the ESG group. At six months of follow-up, the percentage of total body weight loss was much lower in the ESG group than the LSG population, standing at 17.1% versus 23.6%, but the overall adverse events were reported more in the LSG group at 16.9% than the ESG group at 5.2%. Also, new-onset GERD was found to be significantly higher in the LSG group at 14.5% than in the ESG group at 1.9% [6].

Additional scholarly attention was directed at a meta-analysis aimed at evaluating ESG’s safety and efficacy among people diagnosed with moderate to severe obesity [8]. With the follow-up duration being one month, six months, and 12 months, and the results compared with the case of LSG implementation from leading conference proceedings and databases as the focal data sources. Notably, the dependent variables under examination were the BMI, the percentage of excess weight loss, and the percentage of total weight loss. In the findings, at 12 months, the percentage of total weight loss, the percentage of excess weight loss, and the BMI values in the ESG group stood at 17.1%, 63%, and 32.6%, respectively, while the pooled rates in the LSG group at 12 months for the percentage of total weight loss, the percentage of excess weight loss, and the BMI values were 30.5%, 69.3%, and 29.3%, respectively. Therefore, the superiority of LSG’s effectiveness over ESG has been ascertained. When it comes to adverse events, however, the pooled rate associated with LSG was 11.8% compared to 2.9% for the ESG group, suggesting the superiority of ESG in terms of safety. Here, GERD and bleeding events were reported as the leading adverse events [8].

In other scholarly efforts, the follow-up period was 12 months, and the dependent variables on the focus included excess body weight loss and the percentage of total body weight loss. In the ESG groups, the results demonstrated that the values for these variables stood at 60% and 16%, respectively. Some of the factors documented to foster weight loss in the ESG group were affirmed to include post-procedure care in terms of a multidisciplinary team approach and also compliance with regular monitoring. The rate of occurrence of adverse events in the ESG group was found to range from 1.5% to 2.3%, with the new-onset GERD incidence rate deemed negligible. Thus, ESG’s safety profile remained superior, concurring with most of the earlier studies. However, from an effectiveness perspective, LSG retained a superior profile [9]. In a quest to shed more light on this subject, a prospective cohort study was conducted, and results were reported at baseline, six months, and 12 months follow-up [10]. The primary outcome involved the percentage of excess weight loss, with secondary outcomes including adverse events, weight-related quality of life, and body composition. The ESG participants were 16%, while the LSG participants were 45%. In the results, at 12 months post-procedure, values of 57% and 79% were reported as the percent excess weight loss for the ESG and LSG groups, respectively. Improvements in the quality of life yielded 19.8% in the ESG group and 48.1% in the LSG group. However, there was a fat-free mass decrease at six months in the LSG group, but in the ESG group, the fat-free mass was maintained. At 12 months, both groups were confirmed to lose fat-free mass. In terms of safety, the LSG group exhibited 27% of mild-to-severe adverse events, while the ESG group was associated with 25% of mild-to-moderate adverse events [10].

Probing further, another study, conducted from a prospective cohort investigation perspective, compared ESG and LSG performances. Some of the dependent variables that were focused on include quality of life and glycemic biomarkers, as well as adverse events, which were recorded pre-operatively and also two weeks post-operation, with the latter period being the follow-up duration. The number of participants was 50, with 25 exposed to ESG and 25 exposed to LSG. In the results, the abdominal pain was worse in the LSG group compared to the ESG group. These findings are consistent with the findings in the previous studies, which concluded that adverse events occur more in the LSG group compared to the ESG group [12]. In addition, a recent study aimed at highlighting mortality and morbidity outcomes post-operatively had a follow-up period of 30 days. In this study, individuals aged 18 to 80 were the focus, with a BMI range between 35 kg/m2 and 40 kg/m2. The ESG group consisted of 211 cases, while the LSG group constituted 9,059 cases. In the findings, the length of stay and operative length for ESG and LSG stood at 0.49 days versus 1.43 days and 63.9 versus 69.8 minutes, respectively. Hence, ESG exhibited superior performance. In terms of the odds of adverse events, they were lower for the ESG group compared to the LSG group, maintaining the trend in the previously outlined studies [15]. Another randomized controlled trial was also conducted among subjects with non-alcoholic steatohepatitis (NASH) to discern differences in the safety and efficacy of ESG and LSG. With the number of patients standing at 30 and randomized at 1:1 to the control or experimental group, findings saw LSG emerge superior in terms of its ability to enhance weight reduction and hepatic alteration remission. However, the approach was linked to chronic and acute complications post-operatively. On the other hand, ESG proved safer, especially when applied to patients presenting with mild-to-moderate obesity [16].

A systematic review and meta-analysis also pitting ESG versus LSG were conducted in the recent past to uncover the impact of the procedures on patient outcomes [19]. Key databases that were consulted included Cochrane, EMBASE, and MEDLINE. Five specific studies were reviewed, involving two case-matched cohort studies and three cohort studies that compared ESG’s performance with that of LSG. In terms of the total population, 1,451 participants were in the ESG group, while 203 individuals were in the LSG group. For ESG, at six months follow-up, all investigations were avowed to reveal modest short-term loss of the percentage of total body weight, ranging from 13.7% to 15.2%. The LSG group exhibited superior outcomes on this variable, whereby the percentage of total body weight loss ranged from 23.5% to 23.6% at six months of follow-up, and one paper that reported a 12-month follow-up saw a value of 29.3% obtained as the present total body weight loss. In the ESG group, two papers reporting 18 and 24 months of follow-up reported values of 14.8% and 18.6% as the total body weight loss, respectively. However, the rate of complications in the ESG group ranged from 2% to 2.7%, while in the LSG group, it stood between 9.2% and 16.9%. Overall, ESG exhibited lower short-term weight loss outcomes than LSG, but its safety profile remained superior, being a minimally invasive approach [19]. Lastly, a meta-analysis involving 6,775 individuals was used to compare ESG and LSG’s safety and effectiveness [20]. The key databases that were searched comprehensively included Cochrane, EMBASE, and PubMed, with the exclusion of single studies. Here, seven studies were focused upon, with the ESG group involving 3,413 people and the LSG group having 3,362 people. At six-month and 12-month follow-ups, statistically significant differences were reported in which the ESG group yielded values of 7.48% and 7.63%, respectively, while the LSG group yielded values of 10.44% and 11.31%, respectively, centered on the percentage of total body weight loss. Regarding new-onset GERD occurrence, the risk of adverse events stood at 1.3% for the ESG group and 17.9% for the LSG group. Hence, ESG had fewer adverse events than LSG and came with a stomach-sparing nature and an acceptable safety profile, but it had lower short- and mid-term weight loss values, reflecting its inferiority to LSG regarding effectiveness. Thus, it was recommended that ESG is worth implementing mostly with patients exhibiting mild-to-moderate obesity [20].

Discussion

Whereas the results obtained from the selected articles above point to the existence of mixed outcomes, a central theme that emerges is that the majority of the investigations contend that the safety profile of the ESG procedure is superior to LSG due to fewer adverse events with which it may be associated, but the effectiveness of LSG is documented to be superior, hence more efficacious than ESG. What is worth remembering is that some factors can be seen to play a moderating role in depicting these findings. Some of these factors include the ethnic composition of the research population, the age of the participants, their gender, the severity of the disease, and the duration of the investigation and follow-up. When it comes to effectiveness, ESG can be seen to serve better in situations where the goal of weight reduction is short-term. Also, ESG can be seen to exhibit its effectiveness in situations involving mild-to-moderate severity of the disease. On the other hand, the effectiveness of LSG can be affirmed to be more vivid in long-term situations, implying that the procedure may be preferred if there is a long-term weight management goal, with a particular emphasis on the sustainable management of obesity years post-operatively. A key factor worth remembering is that there is also the factor of patient and family preference. On the one hand, ESG as a minimally invasive approach might be highly preferred by patients whose mission is to seek to experience little to no serious adverse effects, including GERD and severe nausea and vomiting. On the other hand, the sustainability of this procedure might not hold, especially in severe cases of obesity requiring long-term therapy. At this point, there is a need for future scholarly studies to center on the key ways in which the divide between patient preferences and physician expertise could be bridged in a quest to ensure the patient’s needs are satisfied while, at the same time, ensuring the adopted procedure comes with a promising degree of effectiveness.

## Conclusions

In summary, ESG and LSG are both promising procedures. However, when considering sustainability and addressing severe obesity cases, LSG can still offer promising outcomes in most situations. It is important to note that factors like age, gender, and ethnicity can influence patients' responses to these procedures. Therefore, an individualized healthcare plan tailored to each patient's specific needs will likely result in the most satisfactory post-operative outcomes for both ESG and LSG.
